# Platelet Metabolites as Candidate Biomarkers in Sepsis Diagnosis and Management Using the Proposed Explainable Artificial Intelligence Approach

**DOI:** 10.3390/jcm13175002

**Published:** 2024-08-23

**Authors:** Fatma Hilal Yagin, Umran Aygun, Abdulmohsen Algarni, Cemil Colak, Fahaid Al-Hashem, Luca Paolo Ardigò

**Affiliations:** 1 Department of Biostatistics and Medical Informatics, Faculty of Medicine, Inonu University, Malatya 44280, Türkiye; cemil.colak@inonu.edu.tr; 2 Department of Anesthesiology and Reanimation, Malatya Yesilyurt Hasan Calık State Hospital, Malatya 44929, Türkiye; umranaygun92@gmail.com; 3 Central Labs, King Khalid University, AlQura’a, Abha, P.O. Box 960, Saudi Arabia; a.algarni@kku.edu.sa; 4 Department of Physiology, College of Medicine, King Khalid University, Abha 61421, Saudi Arabia; fahaid999@yahoo.com; 5 Department of Teacher Education, NLA University College, 0166 Oslo, Norway

**Keywords:** sepsis, platelet metabolomics, biomarkers, machine learning, explainable artificial intelligence

## Abstract

**Background**: Sepsis is characterized by an atypical immune response to infection and is a dangerous health problem leading to significant mortality. Current diagnostic methods exhibit insufficient sensitivity and specificity and require the discovery of precise biomarkers for the early diagnosis and treatment of sepsis. Platelets, known for their hemostatic abilities, also play an important role in immunological responses. This study aims to develop a model integrating machine learning and explainable artificial intelligence (XAI) to identify novel platelet metabolomics markers of sepsis. **Methods**: A total of 39 participants, 25 diagnosed with sepsis and 14 control subjects, were included in the study. The profiles of platelet metabolites were analyzed using quantitative 1H-nuclear magnetic resonance (NMR) technology. Data were processed using the synthetic minority oversampling method (SMOTE)-Tomek to address the issue of class imbalance. In addition, missing data were filled using a technique based on random forests. Three machine learning models, namely extreme gradient boosting (XGBoost), light gradient boosting machine (LightGBM), and kernel tree boosting (KTBoost), were used for sepsis prediction. The models were validated using cross-validation. Clinical annotations of the optimal sepsis prediction model were analyzed using SHapley Additive exPlanations (SHAP), an XAI technique. **Results**: The results showed that the KTBoost model (0.900 accuracy and 0.943 AUC) achieved better performance than the other models in sepsis diagnosis. SHAP results revealed that metabolites such as carnitine, glutamate, and myo-inositol are important biomarkers in sepsis prediction and intuitively explained the prediction decisions of the model. **Conclusion**: Platelet metabolites identified by the KTBoost model and XAI have significant potential for the early diagnosis and monitoring of sepsis and improving patient outcomes.

## 1. Introduction

Sepsis, a life-threatening illness defined by a dysregulated systemic inflammatory response to infection, continues to be a worldwide health concern with catastrophic mortality rates [1]. This acute disease creates excessive and non-specific immune activation, potentially resulting in multiple organ failure and septic shock [2]. Despite continuous improvements in critical care medicine, the intricate biology of sepsis remains incompletely understood. Early identification and the fast start of adequate therapy are crucial for improved survival outcomes [1,2]. However, conventional diagnostic approaches often lack the needed sensitivity and specificity, particularly in the early stages of sepsis [3]. This demands the development of trustworthy biomarkers for early and accurate sepsis diagnosis, disease severity categorization, and therapeutic response tracking.

Clinicians have significant difficulties in diagnosing, treating, and managing patients with sepsis due to its many manifestations. Biomarkers are crucial in the timely detection and classification of sepsis, aiding in the selection of appropriate antibiotics, evaluation of disease severity and prognosis, and assessment of treatment effectiveness. Over 170 biomarker groups have been discovered for evaluating sepsis, encompassing indicators like PCT, CRP, TNF-α/IL-6, MCP-1, and miRNA. However, each biomarker serves a distinct role in the pathophysiology of sepsis, and incorrect utilization of specific biomarkers can result in excessive diagnosis and unnecessary administration of drugs such as antibiotics. As blood cultures have limited ability to detect pathogenic germs, several molecular biodiagnostic disciplines now depend on identifying sepsis by detecting bacterial DNA in the blood. This novel methodology has the potential to result in excessive diagnosis of sepsis by incorrectly recognizing a temporary presence of bacteria in the bloodstream that lacks clinical importance and specific biological traits. There is a pressing need for a novel biomarker to aid in the early detection and treatment of sepsis patients. The evidence demonstrates that during sepsis, the body experiences a condition of heightened metabolism, leading to structural alterations in the three primary nutrients: carbohydrates, proteins, and lipids. However, no one biomarker is considered optimal for diagnosing sepsis or determining its prognosis [4].

Despite the emergence of new technologies, conventional methods remain essential tools for detecting and identifying microbes in sepsis patients. Real-time PCR can identify pathogens in neonatal early-onset sepsis that conventional cultures may miss, potentially enhancing diagnosis and improving treatment outcomes [5]. However, it faces challenges such as high costs, contamination risks, and the inability to detect resistance genes [6]. MALDI-TOF MS is a valuable tool for the rapid identification of bacterial and fungal pathogens in septic patients, providing accurate results in a significantly shorter time frame compared with standard methods [7].

Nevertheless, platelet biomarker-based analysis has the potential to significantly improve the specificity and sensitivity of sepsis diagnosis over conventional methods. By detecting early immune responses, offering greater sensitivity in low-bacterial-load situations, reducing false positives, and providing prognostic information, platelet biomarkers can enhance the speed and accuracy of sepsis diagnosis [8]. This approach is particularly valuable in cases where conventional methods fall short, such as in early or culture-negative sepsis.

Platelets, historically recognized for their hemostatic and thrombotic capabilities, are increasingly acknowledged for their crucial engagement in inflammatory and immunological responses [9]. Platelets have essential functions in fighting infection and are engaged in multiple pathways to enhance the immune response and activate coagulation. Thrombocytopenia is frequently observed in the intensive care unit (ICU) in patients with sepsis. Several factors contribute to this condition, and a low platelet count is associated with a negative prognosis. Gaining a more comprehensive comprehension of the processes that activate platelets and the communication between endothelial cells, immune cells, and pathogens would offer insights into how to specifically target harmful pathways in sepsis, especially those related to platelet activation. Accumulating data show that variations in platelet function contribute considerably to the development and duration of sepsis [10].

Metabolomics, the entire analysis of small-molecule metabolites inside a biological system, offers an effective tool for discovering metabolic irregularities connected with different disorders [11]. Quantitative 1H-nuclear magnetic resonance (NMR) metabolomics has emerged as a particularly promising tool for discovering the distinct metabolic patterns associated with sepsis. This method allows for the comprehensive investigation of a diverse range of metabolites inside platelets, providing a comprehensive understanding of their metabolic state. Through the analysis of these metabolic profiles, researchers may obtain a more comprehensive understanding of the functional changes that occur in platelets during sepsis. Moreover, platelet are better depicted by platelet metabolites rather than total blood metabolites or the interactions of metabolites in biofluids [12].

Metabolomics has been extensively used to investigate the pathogenic processes and biomarkers linked to sepsis. Previous studies have found possible biomarkers that might be used to diagnose sepsis. These biomarkers include metabolites such as 3-phenyl lactic acid, N-phenylacetylglutamine, and phenylethylamine [4]. Recent research has shown that metabolomics analysis may identify distinct metabolic indicators in individuals who do not survive sepsis. Amino acids, mitochondrial metabolism, and eicosanoids are important pathways that are linked to predicting mortality in sepsis cases [13]. Moreover, the combined analysis of untargeted metabolomics and proteomics has revealed abnormalities in inflammation-related pathways and amino acid metabolism as significant factors in sepsis. This offers valuable information about the underlying mechanisms and potential treatment targets for this condition [14]. In a modeling search in the literature at the multi-omics level, including metabolomics, the authors reported sepsis-associated biomarkers, including neutrophil CD10, PTX3, and lysoPC [15]. McBride et al. reported in a review that sepsis-induced mitochondrial dysfunction in leukocytes leads to increased susceptibility to secondary infections in septic patients [16]. Mickiewicz et al. identified 186 metabolites using NMR technology using serum samples from their patients in the ICU. This suggests that metabolomics can predict death in cases of septic shock. In addition, a separate investigation discovered two metabolites that may distinguish between severe sepsis and systemic inflammatory response syndrome. The results emphasize the importance of metabolomics data in predicting and comprehending sepsis outcomes [17,18].

Explainable artificial intelligence (XAI) is a technique used to understand the decision-making process of sophisticated machine-learning models, which are often referred to as black boxes. XAI is particularly effective in analyzing high-dimensional data, such as metabolomics, and it offers improved abilities in generalization and differentiation. This is especially valuable in assessing patient health and identifying issues. The utilization of XAI-based models can not only clarify previously ambiguous biomarkers, but also substantially enhance diagnostic sensitivity and lead to more efficient, individualized treatment options [19].

Although the existing literature has investigated biomarkers of sepsis using metabolomics data and developed predictive models for sepsis detection, no studies have integrated XAI approaches with a specific focus on platelet metabolomics. This study aims to develop an interpretable predictive model in sepsis by identifying candidate metabolomics biomarker compounds derived from platelets based on XAI that may be indicative of sepsis. We strive to study the biological mechanisms underlying sepsis and improve clinical applications by creating a transparent and robust model with a hybrid methodology based on XAI. The results of this research may enable timely and precise antimicrobial treatments and early interventions, ultimately improving patient outcomes and facilitating the use of identified biomarkers in clinical settings to reduce sepsis-related mortality rates.

### Literature Review

The study by McCann et al. [12], using the relevant dataset, used metabolomics to estimate mitochondrial oxygen consumption rates in sepsis patients with multivariate statistical approaches. The study used a predictive methodology of mitochondrial oxygen consumption in sepsis patients. In the current study, we did not consider mitochondrial function; but, unlike using only traditional statistical methods, integrating ML with XAI has the dual advantage of high prediction accuracy and interpretability to distinguish sepsis. We proposed an innovative methodology and developed an explainable prediction model. The inclusion of XAI techniques allowed us to clarify the contribution of individual metabolites to the diagnostic model, thus increasing the clinical relevance and applicability of our findings. Studies have carefully studied the safety and effectiveness of therapies like carnitine or acylcarnitine supplements, which address the metabolic abnormalities in sepsis. Carnitine plays a vital function in mitochondrial transport and the β-oxidation of long-chain and medium-chain fatty acids. In animal models of endotoxemia, carnitine treatment has been demonstrated to reduce circulation levels of tumor necrosis factor (TNF)-α and boost survival rates [20,21]. Small-scale clinical studies have indicated that carnitine infusion may enhance systemic lipid utilization in sepsis and decrease mortality [22,23,24]. Furthermore, Jennaro et al. indicated that the pharmacokinetic response to high-dose carnitine in patients with septic shock is influenced by renal function, and that pre-treatment metabolites might significantly impact the drug’s effectiveness [25]. Keshani et al. reported that administering a high daily dose of 3000 mg of carnitine could potentially decrease inflammation and oxidative stress, thereby improving mortality rates in critically ill patients with sepsis, owing to its anti-inflammatory and antioxidant properties. Recent research has demonstrated that elevated glutamate concentrations are common in neuronal inflammatory illnesses and may be caused by an activated immune system and microglial responsiveness during neuroinflammation [26,27,28]. Impaired glutamate excitotoxicity promotes central nervous system dysfunction and is linked to a variety of diseases, including Alzheimer’s disease [29], epilepsy [30], ischemic disease [31], and traumatic brain injury [32]. Zhenxing et al. [33] found that ferroptosis production in sepsis-associated encephalopathy (SAE) might cause glutamate excitotoxicity and cell death, which can lead to cognitive and behavioral problems. In this study, we confirmed glutamate levels as a potential biomarker for sepsis using SHAP, an XAI technique. Myo-inositol has been thoroughly explored in numerous medical settings, indicating probable linkages to several health issues. Studies show that myo-inositol may be effective in resolving infertility in both men and women [34]. Additionally, its therapeutic potential has been studied in the treatment of cardiac dysfunction generated by sepsis, a disorder recognized for its high fatality rates [35]. Another research has also focused on the use of myo-inositol in preterm newborns, particularly addressing respiratory distress syndrome (RDS), where inositol levels could indicate the illness severity and affect neonatal outcomes [36]. Moreover, the role of myo-inositol in modifying the PI3K/Akt signaling pathway has shown promise in mitigating septic shock by lowering sepsis-induced cardiac dysfunction, hence emphasizing its power to affect sepsis-related morbidity and mortality [37]. The literature has revealed that septic patients have elevated levels of blood ATP and ADP, together with heightened ATPase/ADPase activities, which are connected to the diagnosis of sepsis and are considered possible indicators [38]. Research indicates that ADP, in addition to its role in the dysregulated inflammatory response characteristic of sepsis, along with purines such as ATP, play critical roles in immune and inflammatory responses during sepsis and septic shock [39,40]. Furthermore, inhibiting PARP, an essential enzyme in cellular processes and inflammatory mediator regulation, has been shown to decrease cell apoptosis and cardiac injury during sepsis, underscoring the significance of ADP-related pathways in the pathophysiology of sepsis [40]. Formate, a molecule involved in energy metabolism, has been identified as an important component in sepsis. A study employing NMR-based metabolomics techniques has indicated that formate levels are changed in septic rats, especially in those that do not survive, indicating its potential as a prognostic marker for sepsis [41]. Furthermore, research on septic patients indicated that formate levels were lowered in calves with diarrhea-induced sepsis, indicating its relevance in separating septic conditions from healthy states [42]. These results demonstrate that formate, along with other metabolites, might be a viable biomarker for the early prognostic evaluation of sepsis, emphasizing its significance in understanding the metabolic effects and pathogenic processes of the condition [13,43].

Studies in the literature are based on classical statistical approaches or machine learning methods when examining sepsis biomarkers. The current study is the first to examine metabolomics biomarkers of sepsis based on XAI. The findings of this study will provide important information based on metabolomics biomarkers, especially for the treatment of sepsis in addition to its detection.

## 2. Materials and Methods

### 2.1. Study Design and Participants

The open access data used in this study are available on the NIH Joint Fund’s National Metabolomics Data Repository (NMDR) website, Metabolomics Workbench (www.metabolomicsworkbench.org), where the project ID is designated as ST001294. The Inonu University Health Sciences Non-Interventional Clinical Research Ethics Committee approved this study (approval number: 2024/6097). A total of 39 cases, 25 sepsis patients and 14 controls, were evaluated in this study. The necessary sample size for this investigation was determined using MetSizeR (https://cran.r-project.org/web/packages/MetSizeR/index.html accessed on 8 December 2023), utilizing the probabilistic principal component analysis (PPCA) model. The calculation was performed by specifying a false discovery rate of 0.05. Consequently, it was determined that a minimum sample size of 14 patients, with 7 patients in each group, was necessary. Although it was challenging to recruit patients with SRC and healthy controls who satisfied the specific inclusion criteria outlined in this investigation, the sample size surpassed the estimate generated using MetSizeR [A], a method commonly employed to evaluate sample size in metabolomics studies [44]. This study examined the importance of isolated platelet metabolites in predicting sepsis using quantitative 1H-NMR technology. Sepsis and non-sepsis control patients who were admitted to the emergency department (ED) were included in the study. Platelet samples were collected from each participant at a single time in the ED. The control group was selected to match the sepsis cohort in terms of gender and age [12,45]. The median sequential organ failure assessment (SOFA) score in the sepsis group was 4.5 (3.0–8.5).

#### Inclusion and Exclusion Criteria

− Sepsis Group:
  1. Suspected/proven infection (Organ dysfunction was identified as an acute change SOFA score ≥ 2 points consequent to the infection.) [1].
  2. Any two of the four systemic inflammatory response criteria present in the ED [46].
  3. Must be at least eighteen years old.
  4. At least 2.0 mmol/L of lactate.
  5. Enrollment started two hours after a quantitative resuscitation protocol was started.
− Control Group:
  1. Admitted to the ED.
  2. No medical problems requiring chronic treatment impacting platelet function (e.g., aspirin, P2Y12 inhibitors).
− Exclusion Criteria:
  1. Apart from sepsis as the primary diagnosis.
  2. Declared the status of “Do Not Resuscitate.”
  3. Transfer with previous sepsis treatment from another hospital.
  4. Cardiopulmonary resuscitation before enrollment.

### 2.2. Sample Collection

Whole blood samples (12 mL each) were drawn into tubes containing K2 EDTA using an indwelling line or by direct venipuncture. Centrifugation was used in two steps to remove platelets:

1. Centrifugation at 200× *g* for 6 min at room temperature to separate platelet-rich plasma.
2. A further centrifugation at 4500× *g* for 5 min at 4 °C to pellet the platelets.

After the nearly cell-free plasma was moved to another tube, around 0.25 mL of plasma remained to resuspend the platelet pellet and create ultra-rich plasma. After two freeze–thaw cycles in the presence of methanol, platelet pellets were extracted using methanol and chloroform. This approach ensured the effective extraction of metabolites for NMR analysis. Details regarding platelet isolation, sample extraction for metabolomics, and NMR analyses are included in the Supplementary Materials [1,12,46,47,48,49].

NMR is a spectroscopic method employed to identify organic molecules. NMR spectroscopy can be used to determine the molecular structure of metabolites. Unlike most other metabolomics platforms, NMR has the advantage of not being restricted to the investigation of biofluids or tissue extracts. It is highly appropriate to examine undamaged tissues, organs, and other solid or semisolid samples using solid-state NMR and magic angle sample spinning. This technology is capable of capturing NMR spectra of a variety of nuclei, including 1H, 13C, 15N, and 31P, individually or simultaneously. This allows for the examination of distinct categories of metabolites, such as those containing nitrogen or phosphorus. Multi-dimensional NMR approaches can be used to investigate correlations between two or even three distinct nuclei. NMR spectroscopy is a nondestructive technique that enables easy measurement of fragments without the need for chromatographic separation, sample processing, or chemical derivatization. It facilitates the routine isolation of novel compounds. NMR is extensively automated and very consistent, making it more suitable for high-throughput, large-variance metabolomics research compared with liquid chromatography–mass spectrometry (LC-MS) or gas chromatography–MS (GC-MS). Furthermore, NMR is especially well-suited for identifying and isolating images that are not easily analyzed by LC-MS, such as sugars, organic acids, alcohols, polyols, and other highly polar images [50,51,52,53,54].

### 2.3. Data Preprocessing and Machine Learning Modeling

Missing data were addressed using a random-forest-based imputation approach provided via the miceforest package. This approach effectively imputes missing values by utilizing the links between observable data elements [55]. To handle the issue of class imbalance and prevent biased prediction outputs, the synthetic minority oversampling technique (SMOTE)-Tomek technique was utilized. SMOTE generates synthetic samples for the minority class by interpolating between instances and their nearest neighbors, increasing minority class representation. Tomek links identify and remove pairs of nearest neighbor instances from different classes, cleaning noisy and borderline data points. By first applying SMOTE to balance the class distribution and then using Tomek links to refine the dataset, SMOTE-Tomek achieves improved class balance and clearer decision boundaries [56,57,58]. To guarantee the robustness of sepsis predictions and prevent overfitting, a 5-fold cross-validation approach was performed. This approach separates the dataset into five subgroups, employing four for training and one for validation, rotating through each subset to guarantee full model assessment [59]. In addition to the extreme gradient boosting (XGBoost) and light gradient boosting machine (LightGBM) algorithms for omics data, used due to their efficiency and ability to process high-dimensional data [60,61,62], the kernel-tree boosting (KTBoost) algorithm [63], which offers an important alternative with its hybrid approach and provides potentially good performance in capturing complex patterns, was also used. XGBoost builds an ensemble of decision trees where each tree corrects the errors of its predecessors, incorporating regularization and parallel processing for enhanced performance. LightGBM constructs trees leaf-wise rather than level-wise, optimizing speed and memory usage, and directly handles categorical features [62,64]. KTBoost combines boosting and kernel methods, leveraging the strengths of both to capture complex, non-linear relationships within the data [63]. The aforementioned techniques were chosen because of their capacity to handle extensive feature spaces, scalability, and appropriateness for high-dimensional omics datasets, guaranteeing resilient and precise predictive modeling. We used a number of indicators to assess the effectiveness of the XGBoost, LightGBM, and KTBoost models. The percentage of true positives and true negatives among all the cases examined that were accurately predicted is known as accuracy. Specificity measures the model’s ability to reliably detect negative cases, while sensitivity, often known as recall, measures the model’s ability to properly identify positive situations. AUC, which measures the model’s performance across all classification thresholds, is another useful tool that offers a thorough evaluation of the discriminatory capacity of the model [65,66].

### 2.4. Explainable Artificial Intelligence Approach and Interpretation of Sepsis Biomarkers and Prediction

**Figure 1:**
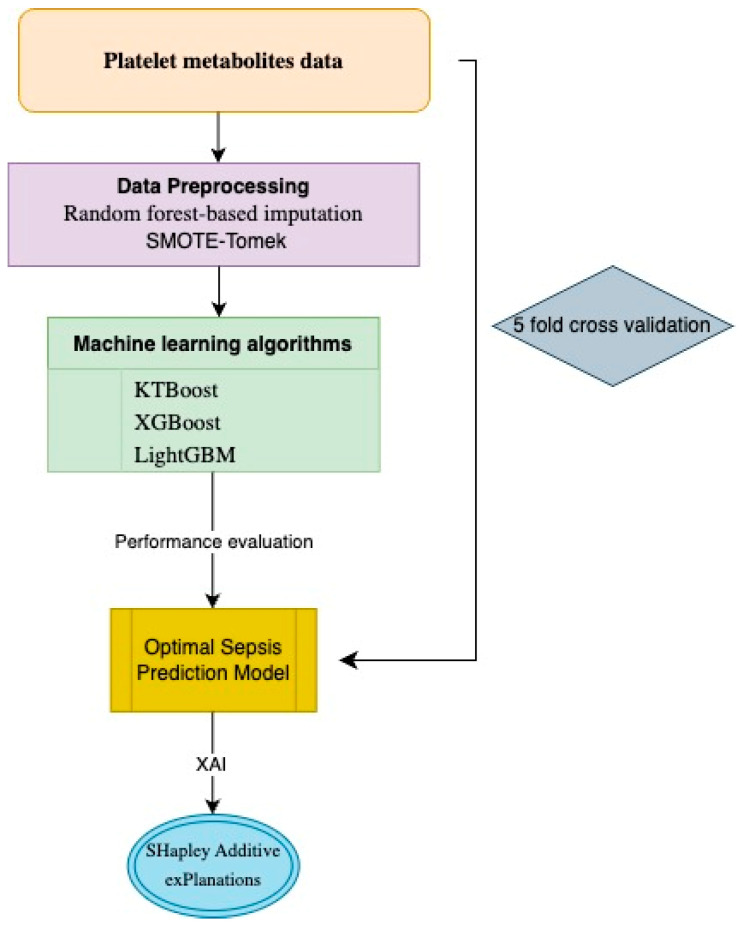
Flow chart of the proposed methodology.

We used the XAI approach to understand the prediction judgments produced by the models. XAI seeks to provide transparency and reliability in AI applications by making the sophisticated machine learning models’ choices and behavior understandable to people. XAI techniques can find important traits, expose biases, and improve the dependability of AI systems by offering insights into how models create predictions. This is especially useful in vital domains like bioinformatics and healthcare. Based on this information, the SHapley Additive exPlanations (SHAP) method was applied to determine the importance of biomarker candidate metabolites in predicting sepsis and to explain the outputs of the model from a clinical perspective. SHAP values are based on cooperative game theory and provide a unified measure of feature importance by considering the contribution of each feature to the prediction. This technique determines the average marginal contribution of each characteristic over all possible combinations, guaranteeing a consistent and fair assignment of significance. By displaying SHAP values, we can comprehend the influence of each feature on individual predictions and the entire model, providing a better understanding of the model’s decision-making process and boosting interpretability [67,68,69,70]. The flow chart for the proposed methodology is presented in Figure 1.

### 2.5. Biostatistical Analysis

Normal distribution was evaluated with the Kolmogorov–Smirnov test. Normally distributed quantitative data were summarized using the mean and standard deviation (SD), and non-normally distributed quantitative data were summarized using the median and interquartile range (IQR). The existence of a statistically significant difference in terms of input variables and the relationship between the “positive” and “negative” groups, which are the categories of the output variable, were examined using the *t*-test and Mann–Whitney U test in independent groups, respectively, for normally distributed and non-distributed quantitative data. For the concentration levels of the five most significant metabolites identified by the XAI method, they are presented in boxplots along with the median and interquartile range. In correlation analyses, the Spearman rho coefficient was calculated to examine the relationships between quantitative variables that did not meet the parametric conditions (normal distribution). *p* values < 0.05 were considered statistically significant. American Psychological Association (APA) 6.0 style was used to report statistical differences. All statistical analyses were performed using IBM SPSS Statistics for Windows version 28.0 (New York, NY, USA) software and Python version 3.9 software.

## 3. Results

Table 1 outlines the characteristics of the participants in the study, detailing their gender distribution and ages. Among men, the control group comprised 8 individuals (57.1%), while the sepsis group included 15 individuals (60%). The difference in representation between the two groups did not show significance (*p* = 0.862). In terms of women, the control group had 6 individuals (42.9%) whereas the sepsis group had 10 individuals (40%). The average age of those in the control group was 44 years (with a deviation of 14), while those in the sepsis group had an age of 55 years (with a standard deviation of 17). The age gap between the groups neared significance, with a *p*-value recorded at 0.056.

Table 2 presents a comparative univariate analysis of metabolite levels between the control and sepsis groups, summarized by median and IQR. While several metabolites exhibit notable trends, few show statistically significant differences. ADP and carnitine levels trend toward lower values in the sepsis group, with near-significant *p*-values of 0.058 and 0.067, respectively. Conversely, O-acetylcholine (*p* = 0.027) and O-phosphoethanolamine (*p* = 0.047) levels are significantly lower in the sepsis group, highlighting their potential as biomarkers for sepsis. The other metabolites, AMP, ATP, alanine, choline, creatine, formate, GTP, glucose, glutamate, glutamine, glycine, lactate, O-phosphocholine, taurine, and Myo-inositol, display no significant differences between the groups (*p* > 0.05) (Table 2).

Table 3 compares the performance of three classification models—KTBoost, XGBoost, and LightGBM—in predicting sepsis, evaluated through accuracy, F1-score, sensitivity, specificity, and AUC. KTBoost demonstrates the strongest performance across all metrics, with the highest accuracy (0.900), F1-score (0.894), sensitivity (0.840), specificity (0.960), and AUC (0.943). XGBoost consistently ranks second, with an accuracy of 0.860, F1-score of 0.851, sensitivity of 0.800, specificity of 0.920, and AUC of 0.914. LightGBM shows the lowest performance, with an accuracy of 0.800, F1-score of 0.792, sensitivity of 0.760, specificity of 0.840, and AUC of 0.861. Although few significant differences in metabolite levels were detected between sepsis and control in the univariate statistical approach, KTBoost, a machine-learning-based multivariate classification approach, showed strong performance in distinguishing sepsis from healthy controls. These results indicate that KTBoost is the most effective model for sepsis prediction, achieving the best balance between accurately identifying true positive and negative cases (Table 3).

**Figure 2:**
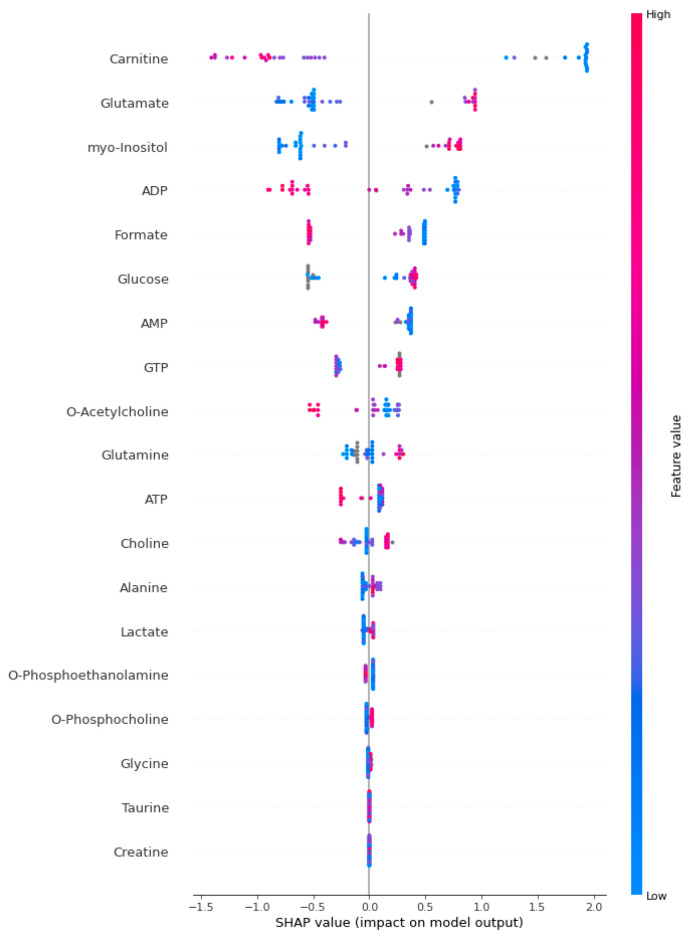
The SHAP summary plot for the KTBoost model. A higher SHAP value indicates a higher likelihood of sepsis occurring. The color of the dots represents the value of the feature, with red indicating high feature values and blue indicating low feature values.

**Figure 3:**
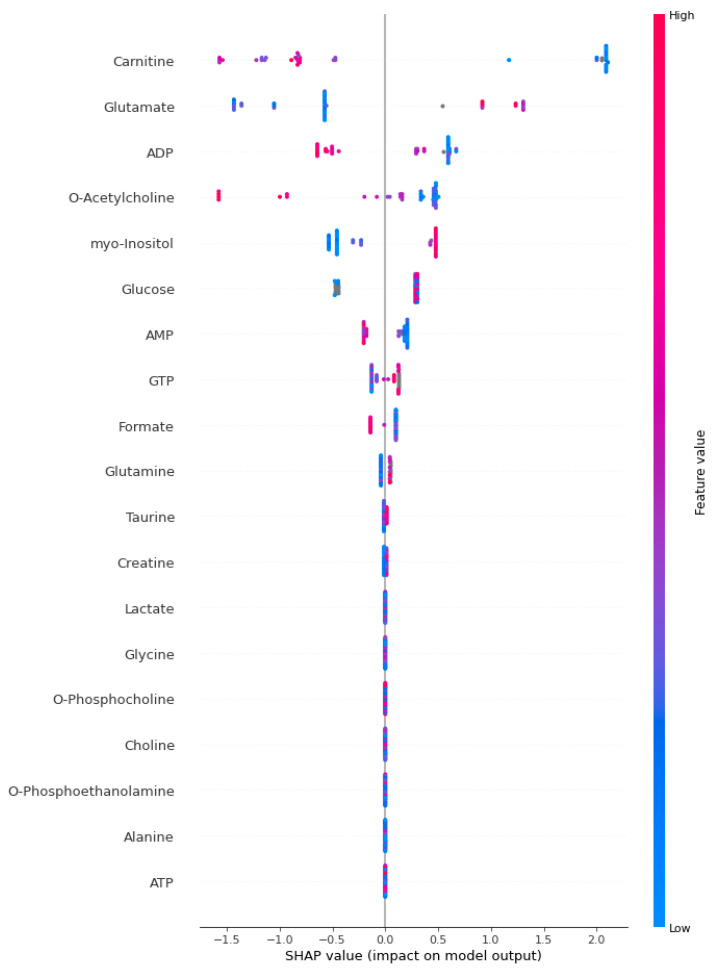
The SHAP summary plot for the XGBoost model.

**Figure 4:**
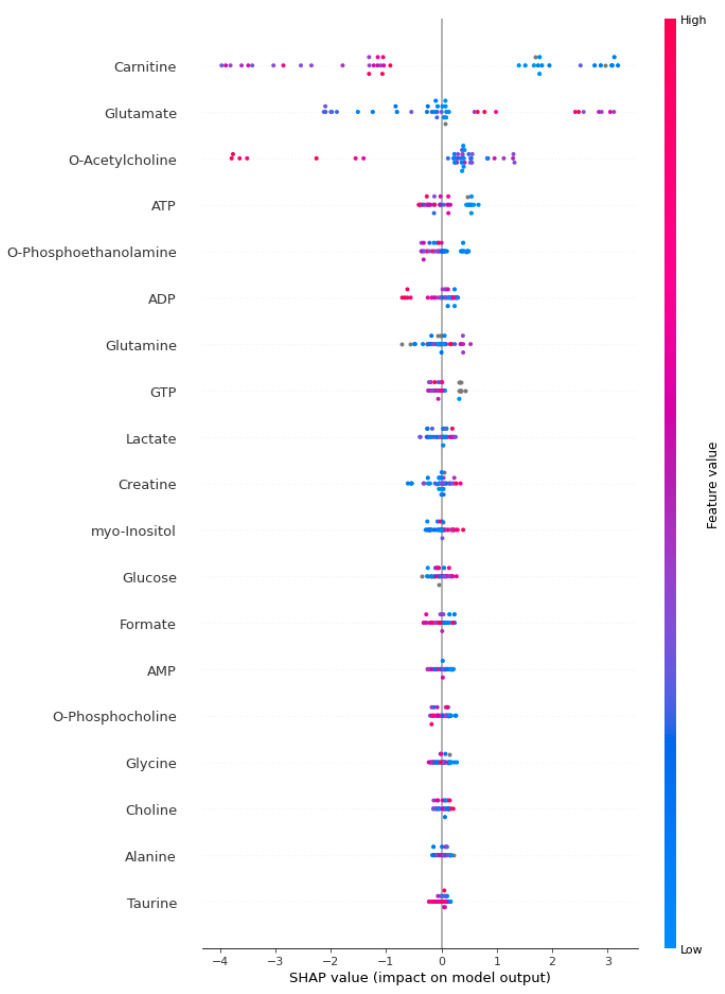
The SHAP summary plot for the LightGBM model.

Figure 2, Figure 3 and Figure 4 present the SHAP summary plot for the KTBoost, XGBoost, and LightGBM models, respectively, describing the impact of biomarker candidate metabolites on sepsis prediction. The X-axis represents SHAP values, which indicate how much each metabolite contributes to the model’s prediction. Positive SHAP values mean that the metabolite increases the likelihood of sepsis, while negative SHAP values mean it decreases the likelihood. The SHAP plot was created to examine the effect of biomarkers on the risk of positive class (sepsis). The colors of the dots in the SHAP summary plot represent the value of the metabolite, with red indicating high metabolite values and blue indicating low metabolite values. Each dot in the plot corresponds to an individual instance (a patient) within the dataset, and the position of the dot along the X-axis indicates the SHAP value for a specific metabolite in that instance. Specifically, in this context, each dot represents one metabolite’s contribution to the model’s prediction for a particular patient. For example, metabolites like carnitine and glutamate show a wide range of SHAP values, indicating their impact on the model’s prediction varies significantly across different instances. Carnitine is a vital metabolite essential for fatty acids’ transport into mitochondria, where they undergo beta-oxidation to generate energy. In sepsis, marked fluctuations in carnitine levels may reflect disturbances in energy metabolism and mitochondrial function, both critical components of the host’s response to severe infection. The significant difference in carnitine levels between septic patients and healthy individuals underscores the disruption of energy metabolism in the complex pathophysiology of sepsis. This disparity likely reflects mitochondrial dysfunction and energy deficiency, which are central to the organ dysfunction characteristic of sepsis.

This plot highlights the metabolites with the highest influence on the model, providing insights into their roles in sepsis prediction and emphasizing the variability in their contributions among the patients. When evaluated with comprehensive performance measures, KTBoost was chosen as the optimal prediction model because it achieved the best performance in sepsis prediction. According to the KTBoost model SHAP annotations, it was determined that low levels of glutamate, myo-Inositol, glucose, GTP, and glutamine metabolites, in addition to low levels of carnitine, ADP, formate, AMP, and O-acetylcholine metabolites, increased the risk of sepsis.

**Figure 5:**
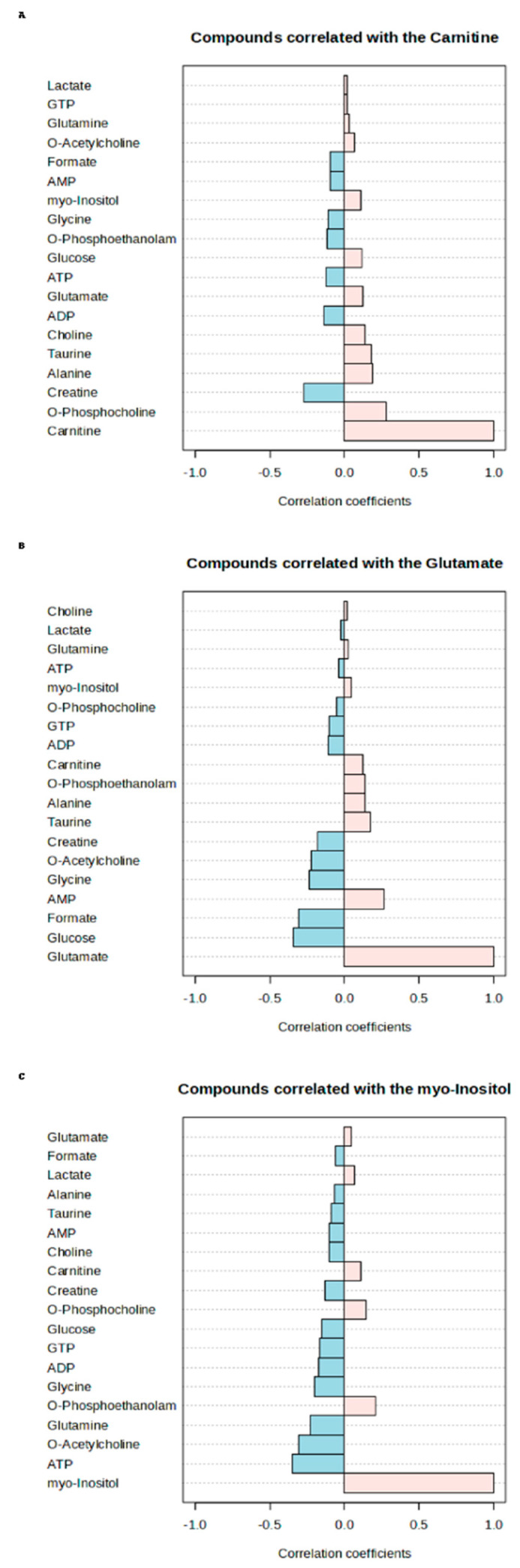
Spearman rho correlation graphs showing the relationship of the three most important biomarker candidate metabolites in sepsis with other metabolites according to SHAP annotations. Graphs are drawn from top to bottom for carnitine (**A**), glutamate (**B**), and myo-inositol (**C**). Pink: positive correlation; Blue: negative correlation.

Figure 5 presents the correlation graphs expressing the relationships between the three most important biomarker candidate metabolites for sepsis (carnitine, glutamate, and myo-inositol) and other metabolites. Since parametric conditions (normal distribution) could not be provided, the Spearman rho coefficient was calculated to examine the relationships between the metabolites. The results show that carnitine has a positive correlation with compounds such as O-phosphocholine, creatine, and alanine, and a negative correlation with lactate and GTP. It highlights that glutamate is positively correlated with glucose, formate, and glycine but negatively correlated with choline and lactate. Furthermore, it shows that myo-inositol is positively correlated with ATP, O-acetylcholine, and glutamine and negatively correlated with glutamate and formate. Correlation plots highlight the interconnected roles of biomarker candidate metabolites in sepsis, which is important for understanding their interactions and potential effects on sepsis pathophysiology at a more molecular level (Figure 5).

## 4. Discussion

Sepsis is a systemic inflammatory response syndrome induced by the infiltration of bacteria and other pathogenic microorganisms into the bloodstream. It often occurs as a secondary complication to other severe illnesses and significant infections of organs or tissues. Currently, there are no reliable biomarkers for the early detection and diagnosis of sepsis, which can rapidly become fatal, making the development of new identification methods critical [71]. Recent models, both in use and under development, based on patient vital signs and routine clinical and laboratory data, have shown promising results for the early detection and prediction of sepsis [72,73]. Studies on gene expression, inflammatory responses, and metabolomics in non-routine datasets have further enhanced these analyses, providing significant insights into treatments such as steroids [74,75,76,77].

In this study, we evaluated platelet metabolomics panel data from patients with sepsis and healthy individuals to identify platelet metabolomics biomarkers with diagnostic potential. Platelets play crucial roles in combating infection and contribute to various pathways that bolster the immune response and trigger coagulation. Thrombocytopenia is a common occurrence in ICU patients with sepsis. In addition, platelets are more accurately represented by platelet metabolites rather than total blood metabolites and the components of metabolites in biofluids [7]. Thus, the present study was centered on platelet metabolomics. To our knowledge, this is the first study to examine platelet metabolomics biomarkers in sepsis patients using XAI approaches. Following extensive data preprocessing, we employed XGBoost and LightGBM, two high-performing tree-based algorithms for omics data, as well as KTBoost, a novel approach combining kernel and tree methods. The KTBoost model outperformed the other models in sepsis detection, achieving sensitivity (0.840), specificity (0.960), and AUC (0.943) values. High sensitivity indicates a low false negative (FN) rate. False positive and false negative errors are common in comparative biological research; thus, determining the likelihood of a true effect being significant is crucial. A lower FN rate is particularly important in sepsis cases, as minimizing missed diagnoses (false negatives) is a primary objective of this research.

Using SHAP values and SHAP plots, we demonstrate that our approach effectively highlights the key features and interprets the machine learning results. The SHAP analysis reveals that the ten most significant biomarker candidate platelet metabolites associated with sepsis and crucial for the model’s decision are glutamate, myo-inositol, glucose, GTP, glutamine, carnitine, ADP, formate, AMP, and O-acetylcholine metabolites. SHAP values measure the relevance of each output by evaluating all feature combinations, delivering consistent and locally accurate values for each feature in the prediction model. This annotation approach is used with KTBoost’s black-box tree-integration model, enabling users to better understand the algorithm’s decision-making process. The precise information on biomarker candidate metabolites received from the findings and annotations boosts doctors’ faith in the algorithm or model and assists in making more informed judgments. Furthermore, domain-specific cumulative feature importance and visualized explanations of feature significance help physicians’ intuitive grasp of the KTBoost model’s essential features and its prediction results. Our approach, given key platelet metabolomics biomarkers, can intuitively explain to clinicians which specific characteristics of sepsis patients increase their disease risk. This predictive approach holds potential in clinical practice by personalizing disease prevention and improving treatment strategies.

The research has shown the significant potential of machine learning approaches in predicting sepsis outcomes when applied to omics data. According to reports, the most effective machine learning model for predicting transcriptomics and sepsis risks is the combination of CatBoost and SHAP [78]. Furthermore, accurate predictions of sepsis survival were made possible by combining metabolomics data with machine learning techniques like support vector machine, naive Bayes, k-nearest neighbors, decision tree, random forest, and artificial neural networks. Of these, artificial neural networks demonstrated the highest accuracy rates [79]. However, these studies were not based on platelet metabolomics biomarkers and XAI for the interpretable prediction of sepsis.

The observed connections among metabolites in our research, including those involving glutamate, carnitine, and myo-inositol, indicate a synchronized metabolic response that is closely linked to the underlying causes of sepsis. These metabolites play essential roles in vital processes like energy metabolism, oxidative stress, and immunological signaling, all of which are disturbed during sepsis. For example, increased levels of glutamate are related to the inflammation of neurons and encephalopathy associated with sepsis, whereas carnitine plays a crucial role in the functioning of mitochondria, which is a major area of malfunction in individuals with sepsis. The relationships seen among these metabolite families are not just accidental, but rather provide an indication of the intricate metabolic network that is responsible for sepsis pathophysiology. Gaining an understanding of these linkages allows us to place these biomarkers in the larger context of sepsis processes, emphasizing the possibility of developing more focused diagnostic and treatment approaches [80].

Tang et al. (2018) utilized a community method to find biomarkers that might enhance mortality forecasts by evaluating gene expression patterns. Cano-Gamez et al. (2022) established a stratified immune dysfunction score based on whole-blood gene expression for acute infection patients. This score predicts patient outcomes and shows how immune system dysregulation progresses sepsis. These papers demonstrate the usefulness of gene expression analysis to improve sepsis prognosis and therapy [81,82].

Our study has a few limitations. First, this is a single-center study, and specific metabolites should be investigated further and confirmed in multicenter trials before widespread implementation. The study’s cross-sectional approach hampered its ability to assess time-dependent platelet metabolomics alterations. Future research may look into the effects of these metabolites on sepsis throughout time. Another limitation was that the control group was small compared with the sepsis group, and in this case, synthetic samples were generated for the control group with the SMOTE-Tomek approach to avoid biased results of the ML approaches. The results can be strengthened with more control samples in future studies. Another limitation is that the study did not differentiate between severe, moderate, and mild sepsis. Although our current model was designed to differentiate between septic and non-septic (healthy controls) patients, the potential for further stratification of sepsis severity should be investigated in future studies. In addition, for platelet metabolite analysis, concentrations in the range of 107 to 108 platelets per sample are often necessary. This equates to a volume of around 500 μL to 1 mL of platelet-rich plasma (PRP) or platelet lysates. While there is no strict threshold for the minimum platelet concentration, ensuring a high enough concentration to achieve a good result is critical for effective NMR analysis and reliable machine-learning model performance. Low platelet levels can introduce significant challenges, including poor data quality, model overfitting, and biased metabolite detection. In the current study, the results related to inflammatory cytokines such as IL-1B, IL-18, IL-10, p-selectin, PF4, and RANTES were not examined, and the effect of platelet metabolomics was examined. In the future, studies using data related to these cytokines together with omics technologies are needed. Future studies will aim to include a larger cohort with groups stratified according to sepsis severity. This will include detailed clinical data collection to accurately classify patients into mild, moderate, and severe sepsis categories. We plan to further develop our model to distinguish between different sepsis severities by integrating additional clinical parameters and expanding the metabolomics dataset. This will involve advanced ML techniques and possibly integration of other omics data (proteomics, transcriptomics) to capture a more comprehensive biosignature of sepsis severity.

## 5. Conclusions

This research offers many important future applications. Combining these applications with routine clinical and biological information can increase early diagnosis and accuracy, improve clinical outcomes, suggest physiological pathways and treatment targets, inform targeted clinical trial participation, and optimize clinical management. In conclusion, XAI is promising for its potential to better target the response to sepsis through precision medicine.

## Figures and Tables

**Table 1:** Patient demographic characteristics of the study participants.

Variable		Group		*p*-Value
		Control	Sepsis	
Gender *	Male	8 (57.1)	15 (60)	0.862
	Female	6 (42.9)	10 (40)	
Age **		44 (14)	55 (17)	0.056

*: The variable is summarized as frequency (percentage), and the chi-square test was used; **: The variable is summarized as mean (standard deviation, SD) and the *t*-test was used in independent groups.

**Table 2:** Comparative analysis of metabolite level changes between control and sepsis groups.

Metabolite Name *	Group		*p*-Value
	Control	Sepsis	
ADP	41.95 (16.875)	27.8 (25.8)	0.058
AMP	8.75 (3.825)	7 (3.8)	0.429
ATP	57.2 (29.15)	47.7 (36)	0.183
Alanine	11.9 (5.5)	12.2 (5.1)	0.781
Carnitine	3.33 (1.075)	1.9 (2)	0.067
Choline	4.725 (1.9)	4.1 (3.6)	0.446
Creatine	3.25 (2.225)	4.1 (2.82)	0.379
Formate	19 (6.6)	18 (5.7)	0.693
GTP	16.05 (2.347)	16.93 (5.3)	0.507
Glucose	56.47 (17.65)	56.47 (15.6)	0.426
Glutamate	83.2 (19.225)	85.7 (61.6)	0.988
Glutamine	34.95 (8.68)	36.83 (16.6)	0.669
Glycine	12 (5.35)	8.6 (6.9)	0.183
Lactate	64.5 (19.85)	65.6 (48.4)	0.965
O-acetylcholine	3.95 (2.65)	3.1 (1.9)	0.027
O-phosphocholine	9.55 (4.85)	6.6 (6.1)	0.224
O-phosphoethanolamine	54.435 (17.9)	38.2 (32.3)	0.047
Taurine	578.3 (239.225)	443.2 (344.5)	0.141
myo-Inositol	22.9 (9.05)	32.21 (29.4)	0.377

*: Metabolites are summarized by median with interquartile range (IQR). ADP: adenosine diphosphate; AMP: adenosine monophosphate; ATP: adenosine triphosphate; GTP: guanosine triphosphate

**Table 3:** Performance results of classification models in predicting sepsis.

Metric/Model	KTBoost	XGBoost	LightGBM
Accuracy	0.900 (0.817–0.983)	0.860 (0.764–0.956)	0.800 (0.689–0.911)
F1-score	0.894 (0.808–0.979)	0.851 (0.752–0.950)	0.792 (0.679–0.904)
Sensitivity	0.840 (0.639–0.955)	0.800 (0.593–0.932)	0.760 (0.549–0.906)
Specificity	0.960 (0.796–0.999)	0.920 (0.740–0.990)	0.840 (0.639–0.955)
AUC	0.943 (0.900–0.987)	0.914 (0.860–0.968)	0.861 (0.800–0.923)

AUC: Area under the curve; KTBoost: kernel-tree boosting; XGBoost: extreme gradient boosting; LightGBM: light gradient boosting machine.

## Data Availability

Data can be requested from the corresponding author upon appropriate request.

## Supplementary Materials

The following supporting information can be downloaded at: https://www.mdpi.com/article/10.3390/jcm13175002/s1, Figure S1: Violin chart for Creatine levels; Red: control; Green: Sepsis; Figure S2: Violin chart for glutamate levels; Red: control; Green: Sepsis; Figure S3: Violin chart of myo-Inositol levels; Red: control; Green: Sepsis; Figure S4: Violin chart of ADP levels; Red: control; Green: Sepsis; Figure S5: Violin chart for Formate levels; Red: control; Green: Sepsis. References [12,15,16,17,18,19] are cited in Supplementary Materials.
