# Supplementary material for: Platelet Metabolites as Candidate Biomarkers in Sepsis Diagnosis and Management Using the Proposed Explainable Artificial Intelligence Approach
Source: J Clin Med. 2024 Aug 23;13(17):5002. doi: 10.3390/jcm13175002 (PMC11395774; doi:10.3390/jcm13175002)
Supplement: Supplementary file 1 [file jcm-13-05002-s001.zip › jcm-3163623-supplementary.pdf]

Sample Extraction for Platelet Isolation and Metabolomics

In this study, available metabolomics data were obtained from the Metabolomics Workbench database. To obtain the relevant data, blood (12 mL) was centrifuged for platelet isolation (200× g for 6 min at room temperature). The platelet-rich plasma layer was transferred to another tube and centrifuged to pellet the platelets (4500× g for 5 min at 4 °C). The nearly cell-free plasma layer was transferred to a new tube, leaving approximately 0.25 mL of plasma in the original tube. This remaining plasma was used to resuspend the platelet pellet to produce ultra-rich plasma. Platelets in the ultra-rich plasma were counted with an automated cell counter (Cellometer AutoM10; Nexcelom Bioscience, Lawrence, MA, USA) and diluted with additional plasma as needed to reach a concentration of ~200 × 10^6 cells/mL. Measurements were performed in patient's own plasma instead of buffered media, consistent with previous studies [15]. After isolating and counting platelets as described above, resuspended platelets were centrifuged (4500×g, 5 min, 4 °C), decanted, and then resuspended in 1 mL of methanol (20 °C) in preparation for metabolomic analysis. Cell lysis was performed by snap-freezing samples in liquid nitrogen for 30 s and allowing them to thaw to room temperature before storing at −80 °C. Frozen samples were sent to the laboratory on dry ice for analysis, where they were stored at −80 °C. Immediately prior to analysis, samples were subjected to a second freeze-thaw cycle by snap-freezing in liquid nitrogen and thawing to room temperature [16]. Platelet pellets were kept on ice throughout the extraction period. Samples were transferred to 5 mL centrifuge tubes, and chloroform was added to each resuspended pellet to create a 1:1 methanol:chloroform solution. An additional 250 µL of 1:1 methanol:chloroform was added, followed by 1 mL of DI water, and finally 500 µL of DI water. Samples were vortexed (30 s) after each solvent addition. After the final water addition, samples were vortexed until white and opaque. Samples were cooled in an ice-water bath (15 min), then centrifuged (1000×g, 15 min, 4 °C). After centrifugation, a fine pellet of cellular debris and precipitated protein separated the upper aqueous layer of the extracted sample from the lower chloroform layer. The aqueous supernatant was removed, lyophilized, and resuspended in 50 mM phosphate buffer in deuterium oxide in preparation for NMR [17,18].

Acquisition of Quantitative 1-D-1H-NMR Metabolomics Data

During the experiment to obtain the open-access metabolomics data used in this study, samples were thawed on ice and prepared for NMR analysis as previously described [19]. Sample volume was measured and recorded. An internal standard, sodium 2,2-dimethyl-2-silapentane-5-sulfonate-d6 (DSS-d6), containing 0.2% sodium azide (Chenomx Inc, Edmonton, AB, CA) at a known concentration, was added to each sample. The pH of the sample was measured and corrected to 6.5–7.5 by dropwise addition of 0.1 mM deuterium chloride (CIL) or sodium deuteroxide (CIL). Samples were transferred to 5 mm 800 MHz sensitive NMR tubes (Wilmad Lab Glass, Vineland, NJ, USA) for the experiment. NMR spectra were obtained on an Ascend magnet Bruker

18.8 Tesla (800 MHz) NMR spectrometer equipped with a 5 mm Triple resonance reverse detection TCI cryoprobe and a Bruker NEO console operated with TopSpin 4.0.7 software. The Bruker pulse array noesygppr1d was used to record NMR spectra. Spectra were collected with 128 scans for platelet extracts. The NMR pulse array was as follows: 10 ms recovery, 990 ms saturation pulse, two calibrated 90° pulses, 100 ms mixing time, final 90° pulse, and 4 s acquisition time. The receiver gain was set to 0.5 for all samples. Optimal excitation pulse widths were obtained using a range of pulse lengths as previously described [18, 20]. NMR spectra of platelets were analyzed using Chenomx NMR Suite 8.2 (Edmonton, AB, Canada) software. The processor module was used to phase shift, baseline correct, and water remove from each spectrum as previously described [18, 20]. Compounds were then identified and quantified using the software's Profiler module, which allows metabolites to be quantified against an internal standard of known concentration [18, 20]. All unprofiled peaks were checked to determine if they corresponded to identifiable compounds, in which case they would be profiled. Any volatile compounds (e.g., ethanol) were removed from the dataset prior to analysis, as lyophilization can inconsistently remove these compounds from extracts [18].

Quality Control, Precision, and Accuracy

Quality control was achieved by using a consistent internal standard (DSS-d6) across all samples, allowing for accurate quantification of metabolites. Calibration of the NMR spectrometer and the use of Chenomx software provided high precision in peak identification and quantification. Pulse sequence and receiver gain settings were optimized and standardized across all samples to maintain measurement consistency.

The precision of the measurements was verified by consistent profiling of samples to ensure that the identified compounds were reproducible across samples. Any discrepancies were addressed by re-evaluating the spectra and adjusting the profiling parameters as necessary. The accuracy of metabolite quantification was ensured by calibrating the system using known internal standard concentrations and performing routine checks with quality control samples.

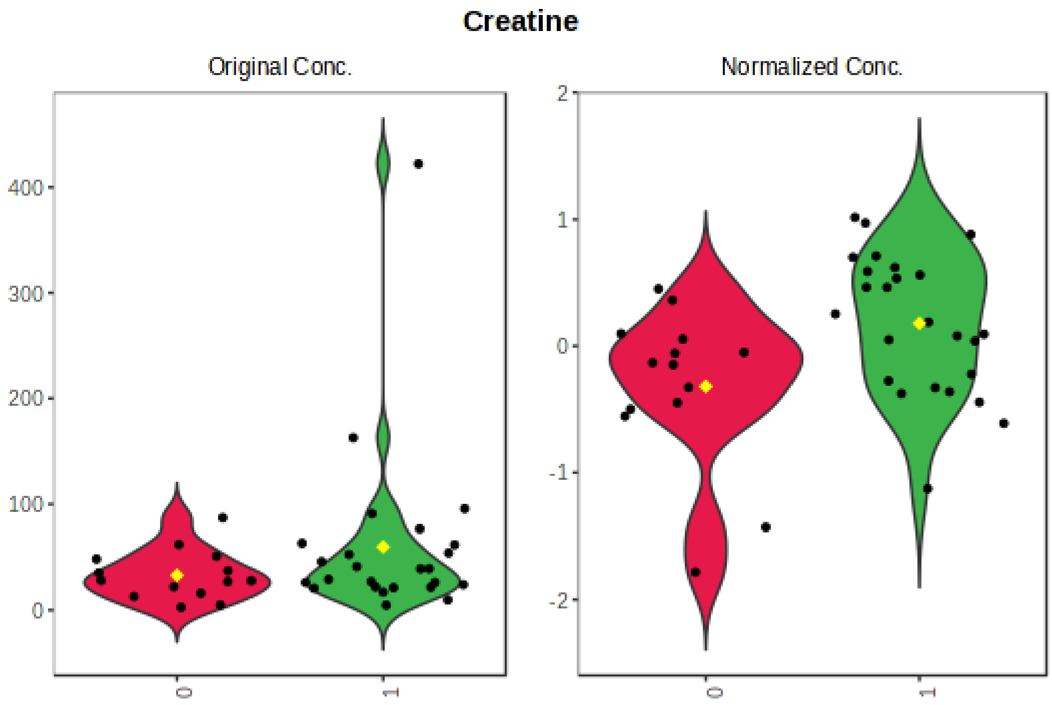

Figure S1. Violin chart for Creatine levels; Red: control; Green: Sepsis.

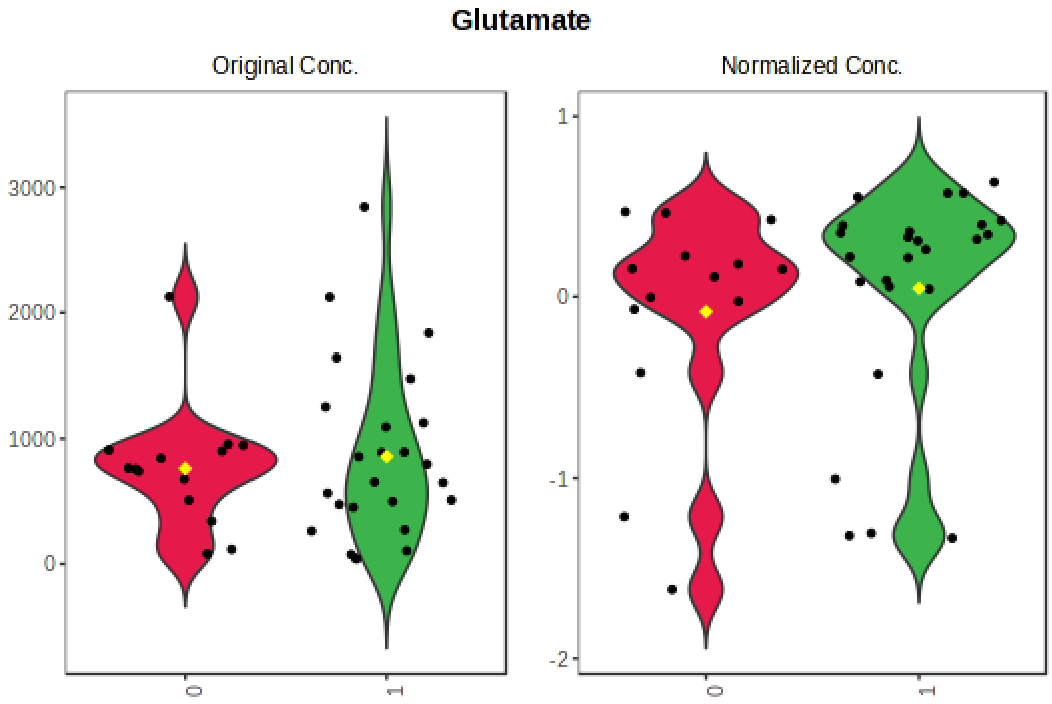

Figure S2. Violin chart for glutamate levels; Red: control; Green: Sepsis.

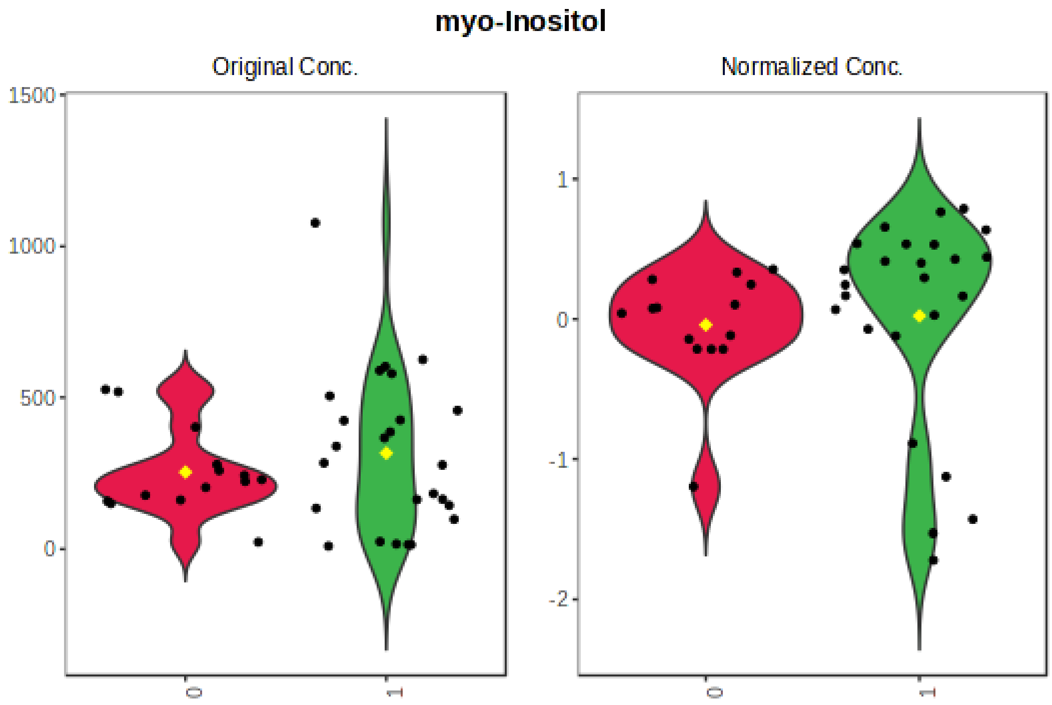

Figure S3. Violin chart of myo-Inositol levels; Red: control; Green: Sepsis.

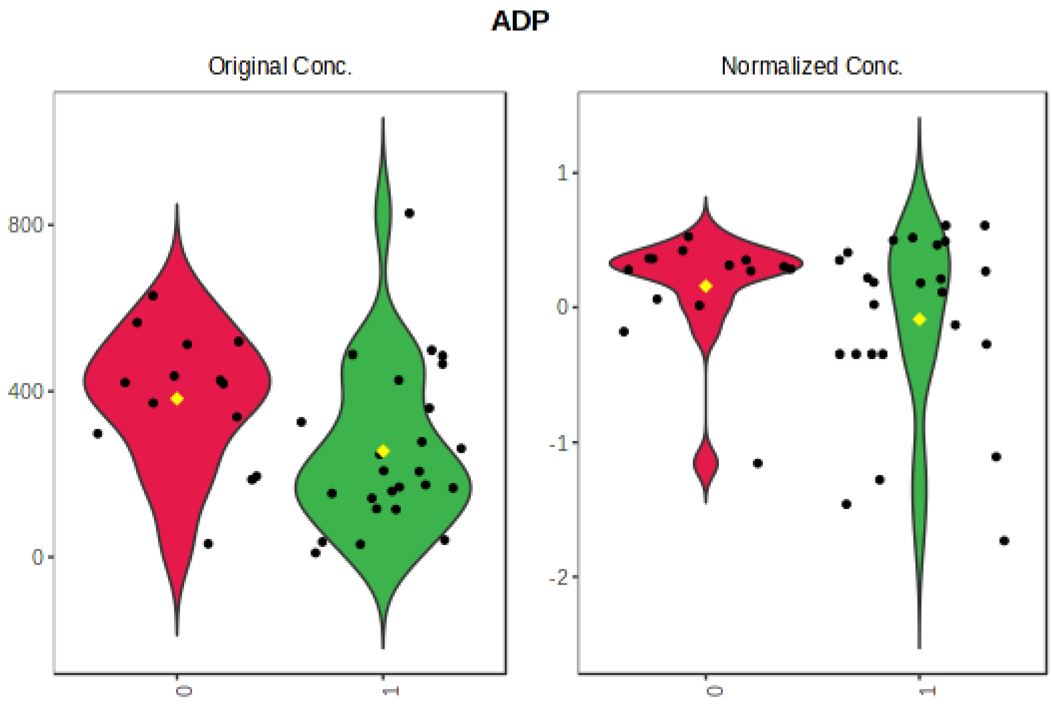

Figure S4. Violin chart of ADP levels; Red: control; Green: Sepsis.

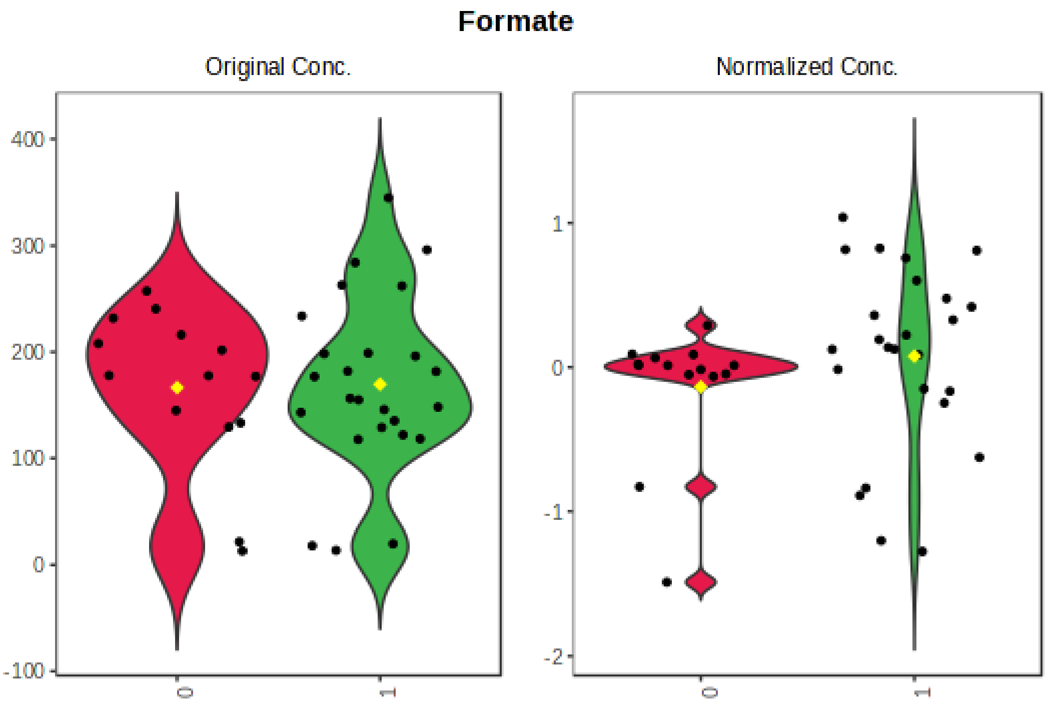

Figure S5. Violin chart for Formate levels; Red: control; Green: Sepsis.
